# Nanostructured Carbon Fibres (NCF): Fabrication and Application in Supercapacitor Electrode

**DOI:** 10.3390/polym16131859

**Published:** 2024-06-28

**Authors:** Kabir O. Oyedotun, Katlego Makgopa, Thabo T. Nkambule, Mkhulu K. Mathe, Kabir O. Otun, Bhekie B. Mamba

**Affiliations:** 1 College of Science, Engineering and Technology, University of South Africa, Florida Science Campus, Private Bag X6, Johannesburg 1709, South Africa; mathemk@unisa.ac.za; 2 Department of Chemistry, Faculty of Science, Tshwane University of Technology, Arcadia Campus, Pretoria 0001, South Africa; makgopak@tut.ac.za; 3 Institute for Nanotechnology and Water Sustainability (iNanoWS), College of Science, Engineering and Technology, University of South Africa, Florida Science Campus, Private Bag X6, Johannesburg 1709, South Africa; nkambtt@unisa.ac.za; 4 Department of Chemistry, College of Science, Engineering and Technology, University of South Africa, Florida Science Campus, Private Bag X6, Johannesburg 1709, South Africa; 61061999@mylife.unisa.ac.za

**Keywords:** carbon nanofibres, polymer, electrodes, electrochemical behaviours, interconnection, supercapacitor

## Abstract

A facile interconnected nanofibre electrode material derived from polybenzimidazol (PBI) was fabricated for a supercapacitor using a centrifugal spinning technique. The PBI solution in a mixture of dimethyl acetamide (DMA) and N, N-dimethylformamide (DMF) was electrospun to an interconnection of fine nanofibres. The as-prepared material was characterised by using various techniques, which include scanning electron microscopy (SEM), X-ray diffractometry (XRD), Raman, X-ray photoelectron spectroscopy (XPS), and Brunauer–Emmett–Teller (BET) among others. The specific surface area of the interconnected NCF material was noticed to be around 49 m^2^ g^−1^. Electrochemical properties of the material prepared as a single-electrode are methodically studied by adopting cyclic voltammetry, electrochemical impedance spectroscopy, and constant-current charge–discharge techniques. A maximum specific capacitance of 78.4 F g^−1^ was observed for the electrode at a specific current of 0.5 A g^−1^ in a 2.5 M KNO_3_ solution. The electrode could also retain 96.7% of its initial capacitance after a 5000 charge–discharge cycles at 5 A g^−1^. The observed capacitance and good cycling stability of the electrode are supported by its specific surface area, pore volume, and conductivity. The results obtained for this material indicate its potential as suitable candidate electrode for supercapacitor application.

## 1. Introduction

The need for alternative energy is now obvious because of global resource depletion and increased energy use. Since the majority of these energy sources need a storage component, electrochemical energy storage devices like batteries and supercapacitors are one of the principal focuses of storage systems for replenishable and green energy sources [1,2,3,4]. As a result of their ability to be replenished, and appropriateness for both mobile and stationary systems, the majority of these combination systems are hitherto employed in new sophisticated technologies to store electricity [5,6].

With the development of hybrid/electric vehicles and small electronic devices, supercapacitors have gained popularity as supportive energy storage technologies and alternatives to batteries due to their record power capacity, proficiency, rapid charging–discharging ability, and remarkable cycling reliability, among other reasons [6,7,8]. The call to advance energy storage systems for various mobile and fixed applications has recently increased interest in studies on batteries, which include lithium-ion batteries [7,8]. However, solutions that are highly effective, economical, rechargeable, eco-friendly, and secure are still required for difficult tasks. It is still debatable if lithium-ion batteries will be able to supply all the energy needs in the future.

The new energy storage technology known as supercapacitors (SCs), sometimes called electrochemical capacitors (ECs), are devices with high power capability, extended cycle life, and strong stability alongside quick charge–discharge speed. They can be employed in a broad range of industries, like fuel cells and electric/hybrid automobiles, remote controls, memory backup systems, solar watches, flashlights, and portable electronic devices, to name a few [9,10,11,12]. Supercapacitor technology is being used in a wider variety of applications, sometimes replacing batteries and other times enhancing their functions [13,14,15,16]. However, because SCs have a lower energy capacity than lithium-ion batteries, their real-world applications are still rather limited [9]. 

Studies on SCs have been centered heavily on the study of advantageous materials with various nanostructures because the electrode materials for SCs are vital to the functioning of the devices. One of the main areas of study in supercapacitors has always been the fabrication of electrode materials with superior electrochemical characteristics [17].

Supercapacitors have been studied using a variety of materials, but their utilisation is still minimal in terms of energy capability [18,19,20,21,22,23,24,25]. Many researchers have recently worked hard to develop and modify carbonaceous materials to increase the specific energy of supercapacitors without sacrificing their power capability. These modifications include modifying the morphology and distribution of pore sizes, adding electroactive metallic particles or electroconductive polymers, and creating hybrid-type devices [25,26]. Supercapacitors can use a variety of carbonaceous materials as electrodes, including powder, fibre, cloth/paper, carbon nanotubes, and related nanocomposites [26,27,28,29].

Using an electric field between two electrodes and an electrostatic repulsive force to create a linked architecture of nanofibres by applying a high potential to a polymer solution is known as electrospinning [28,29]. The wide specific surface area and capacitance are the result of the nano-size diameters. As a result, extensive research has been carried out on the electrospinning of polymer solutions to benefit from a quick, easy, and reasonably priced procedure.

The electrospinning process has recently been used to create carbon nanofibre structures [28,29,30], which may hasten the utilisation of these materials in electrical energy storage devices. Polybenzimidazole (PBI)-based carbon nanofibres have garnered significant attention recently due to their remarkable thermal stability and chemical resistance [28]. These advanced materials are particularly relevant in high-performance applications such as energy storage, filtration, and catalysis. Recent progress in PBI-based carbon nanofibres focuses on enhancing their structural properties and functional performance [29,30]. Innovations include optimising the electrospinning process to produce more uniform and defect-free fibres, incorporating various nanomaterials to improve conductivity and mechanical properties, and exploring post-treatment methods to enhance thermal and chemical stability [27,28,29,30]. These developments are paving the way for PBI-based carbon nanofibres to become integral components in next-generation energy storage technologies.

Because the carbon nanofibres in polybenzimidazol (PBI) have great mechanical strength, a high carbon yield, outstanding thermal stability, and chemical durability, PBI is an excellent choice for creating these kinds of structures. Since PBI-based fibre materials have a thermosetting property derived from the high degree of polymer chain stiffness, they do not require stabilisation, in contrast to precursors of carbon fibres like cellulose, phenol resin, polyacrylonitrile, and pitch, which need a stabilisation procedure before carbonisation/activation to maintain the original form at the carbonisation temperature. The entire production process is determined by the stabilisation stage, which is also the most expensive. Because the PBI-based fibre is simply employed for carbon and activated carbon fibres with carbonisation and/or activation without additional treatment, they can offer a high yield of carbon and save energy. In this study, a PBI-based interconnected carbon fibre material made by spinning and refluxing was assessed as a material for supercapacitor electrodes. The material is made up of uniformly spaced nanofibres that have a specific surface area of 49 m^2^ g^−1^ with the presence of both micro- and mesopores according to the BET analysis. Polybenzimidazol-based carbon nanofibres are an excellent substitute for this application because of their advantageous features, which include large specific surface areas, low electrical resistivity, tunable pore architectures, and high porosities, as well as remarkable electrical and mechanical properties. The novelty of this study lies in the preparation of the material via facile reflux and electrospinning techniques to achieve the interconnection of NCFs as electrodes for supercapacitors. The study reported that the prepared NCF nanofibres, if further investigated and enhanced, could be a potential electrode material in supercapacitor technology. 

## 2. Experimental

### 2.1. Materials

Poly(2,2′-(m-phenylen)-5,5′bibenzimidazol (PBI, molecular weight (MW) = 308.34 g mol^−1^), dimethylacetamide (DMA, MW = 87.12 g mol^−1^), N, N-dimethyl formamide (DMF, MW = 163.15 g mol^−1^), 1-Methyl-2-pyrrolidinone (NMP, MW = 99.13 g mol^−1^; battery grade), conductive carbon acetylene black (CAB; for lithium-ion battery) and polyvinylidene difluoride (HSV900 PVDF, MW = 180,000 g mol^−1^; battery grade) were purchased from Sigma-Aldrich (Inc., St. Louis, MO, USA). All the chemicals in this work were used as purchased and without any further purification.

### 2.2. Synthesis of Carbon Nanofibre 

A total of 20 wt % polybenzimidazol (PBI) was first dissolved in a mixture of dimethylacetamide (DMA) and N, N-dimethylformamide (DMF) at a ratio of 1 (DMA):1(DMF) and refluxed for 6 h whilst stirring at 300 rpm at a temperature 180 °C. Thereafter, the reflux apparatus was allowed to cool down naturally. The polar nature of the added DMA permits it to function as a blended solvent and catalyst in the reaction resulting in a high yield and ideal product in a short term. The resulting mixture was spun into a fibre network via the positively charged capillary utilising an electrospinning machine at DC 20 kV (NanoSpinner, PilotLine PE-300, Innovenso, Cambridge, MA, USA). A 20 cm^3^ syringe with a 0.5 mm diameter capillary tip was filled with the polymer mixture. The syringe tip was clamped to the positive terminal of the high-potential power supply, while the negative terminal was linked to a metal collector. The foil used to collect the electrospun fibre was cloaked around a drum of metal that was rotating at a speed of about 300 rpm. The resulting networked nanofibres sample was then subjected to pyrolysis, which involved heating it to 1000 °C at a rate of 4 °C min^−1^ for 1 h, after previously being heated to 300 °C for 3 h. The adopted two-step heating process at a low ramping rate was to avoid the collapse of the interconnected structure of the nanofibres.

### 2.3. Materials Characterisation

Using a Micromeritics TriStar II 3020 device (Micromeritics, Norcross, GA, USA) controlled at a relative pressure (p/p0) in the span of 0.01–1.0 before being degassed at 110 °C for 20 h, N_2_ absorption–desorption isotherms of the NCF material was obtained. By employing the Brunauer–Emmett–Teller (BET) and Barrett–Joyner–Halenda (BJH) procedures, the sample’s surface area and pore size distributions (PSD) were examined. Morphological examination of the material was performed with a Zeiss Crossbeam 540 FEG scanning electron microscope (FEG-SEM) (Zeiss, Jena, Germany) operated at 2.0 kV, attached with EDX facilities. The sample’s Raman spectrum was captured using a WITec Alpha 300RAS+ confocal micro-Raman imaging system with a 532 nm excitation laser set at a 10.0 mW power for an accumulation period of 120 s. X-ray diffraction investigation was achieved by employing an XPERT-PRO diffractometer operating with cobalt k_α_ radiation at 35 kV and 50 mA. The XRD diffractogram of the sample was measured in the range 10.0–80.0° with 15.24 s counting time, while the XPS of the sample was conducted by using a VG Thermo ESCALAB 250 spectrometer (Thermo Fisher Scientific, Waltham, MA, USA) operating with an Al-Kα exciting source. 

### 2.4. Electrochemical Characterisation 

With EC-Lab VI.41 software and a Bio-Logic VMP300 potentiostat (Knoxville, TN, USA), three-electrode measurements of the as-prepared half-cell were performed. By mixing 80% of the sample as working material, 10% conductive carbon acetylene (CAB) and 10% polyvinylidene difluoride (PVDF) used as a binder in some drops of 1-methyl 2-pyrollidone, the as-synthesised NCF material was fabricated as working electrodes. The added CAB as a conducting agent was to cater to the insulating effect of the PVDF binder in the electrode. The mixture was then stirred for ~120 s in an agate mortar with the aid of a pestle to form a smooth slurry at room temperature. The resulting slurry was then applied on a piece of rectangular nickel foam that had been cut to dimensions of 1 × 1 cm^2^ and 0.2 mm, which serves as a current collector. After that, the electrodes were left in an oven set to 70 °C for the entire night. It was estimated that the electrode’s precise active material loading mass in the half-cell was ~3.5 mg. A 2.5 M KNO_3_ solution was used for all electrochemical studies, while glassy carbon and Ag/AgCl were used as the reference and counter electrode, respectively. The fabricated electrode was subsequently investigated by employing electrochemical impedance spectroscopy (EIS), galvanostatic charge-discharge (GCD), and cyclic voltammetry (CV), respectively.

The following equation was used to estimate the half-cells’ gravimetric specific capacitance, $C_{s}$:

(1) $$C_{s}=\frac{I_{d}\times \Delta t}{∆E}\;\;\;\;\;(F\;g^{-1})$$

where $I_{d}$ is specific current (A g^−1^) of active material, Δ*E* =(${E_{o}-E}_{IR-drop}$) is a change in working potential, E(V) and Δ*t* are discharge time (s) for the single electrode. 

Using the following equation, the coulombic efficiency, *C_E_* of the single electrode was determined over an electrode potential (V):

(2) $$C_{E}=\frac{{C_{s}}_{d}}{{C_{s}}_{c}\;}\times 100\%$$

where ${C_{s}}_{c}\;$and ${C_{s}}_{d}$ represent specific capacitance for the charge and discharge process, independently.

## 3. Results and Discussion

### 3.1. Morphological, Structural, and Compositional Analyses 

**Figure 1:**
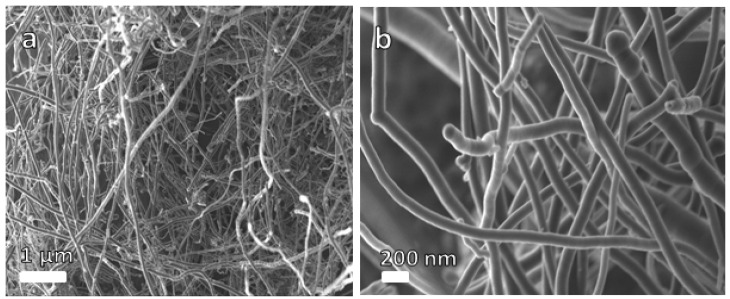
SEM images of carbon nanofibres material at (**a**) low and (**b**) high magnifications, respectively.

Figure 1 shows SEM micrographs of the NCF sample. The SEM pictures of the as-produced carbon nanofibres at different magnifications are exhibited in Figure 1a,b, showing its interconnected nanofibres’ morphology. According to the SEM analysis, the material is made up of uniformly spaced nanofibres with an average diameter of 230 nm.

**Figure 2:**
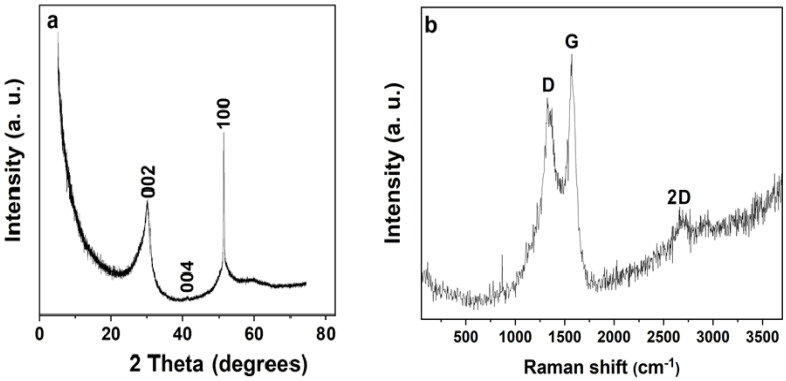
(**a**) XRD, and (**b**) Raman spectrum of NCF sample.

Figure 2a shows the XRD pattern of the sample. The sharp peak at 2θ = 30.3° is comparable to the (002) plane of graphitic carbon [19,20] indexed according to the JCPDS card number: 41-1487. The silica substrate material employed in sample preparation for the analysis is what is responsible for the peak at almost 2θ = 41.3°; (004) plane. The observed sharp peak around 51.3° (100) is an indication of the sp^2^ hybridised carbon structure, suggesting that the networked material fabricated from electrospinning was successfully carbonised after the pyrolysis process. The well-defined D and G bands peak at roughly 1317 and 1568 cm^−1^, separately, in the Raman spectra shown for the material in Figure 2b indicating a feature of carbon material. The observed D and G peaks are attributed, respectively, to structural flaws and in-plane vibration of sp2 carbon atoms. The intensity ratio of the sample’s D-G peak (I_D_/I_G_) was determined to be 0.84 for NCF material. The sample’s recorded high-intensity ratio is an indication of a high degree of disordered carbon, indicating a modest level of defect density in the material. A 2D band peak is seen at almost 2695 cm^−1^, which further supports the crystalline character of the fibre material [21]. 

**Figure 3:**
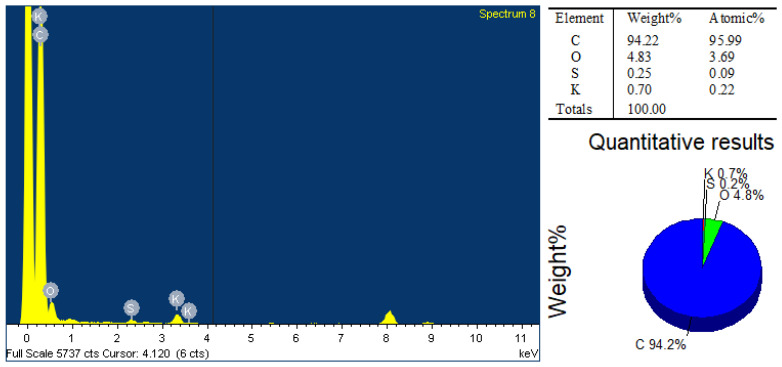
EDX analysis of NCF sample.

The elemental analysis of the NCF sample is shown in Figure 3, which was performed via an EDX device attached to the SEM device. The EDX spectrum along with its elemental table and a pie chart as a representation of its quantitative results as displayed in Figure 3 indicate that carbon and oxygen are the major elements that exist in the nanofibres material. The existence of traces of sulphur and potassium in the EDX spectrum could be ascribed to the sample preparation. 

**Figure 4:**
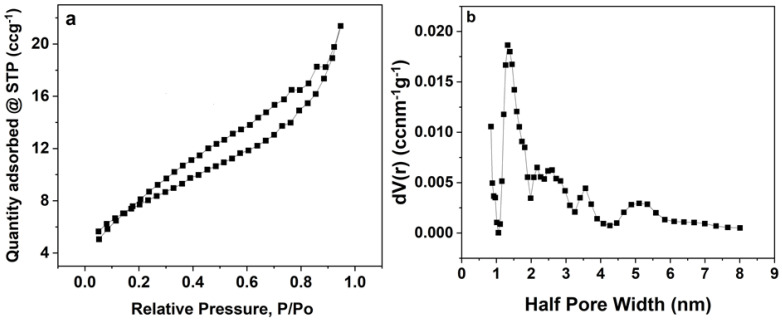
(**a**) N_2_ isotherm, and (**b**) PSD analysis of NCF sample.

A type II N_2_ isotherm of the sample obtained at standard pressure and temperature (STP) is shown in Figure 4a. According to the graph, the complex material that contains both micropores and mesopores is the cause of the H4 hysteresis between the adsorption and desorption isotherms as well as the abrupt decline in the quantity of adsorbed materials at higher relative pressures [18].

Figure 4b presents the material’s average pore size distribution, which was achieved by using the BJH technique. A peak is visible at around 1.5 nm in this distribution. The specific surface area (SSA) of around 49 m^2^ g^−1^, as well as the micropore volume of nearly 0.17 cm^−3^ recorded for the material, can be beneficial for charge storage by the facilitation of good adsorbate accessibility with longer flow routes to micropores. Ions can fit inside the pores of the material due to the sample’s average pore size, which is more than the ions’ average size of the KNO_3_ electrolyte, which is about 0.13 nm. As a result, the process of storing charge is improved as well as the accessibility to the electrolyte. 

**Figure 5:**
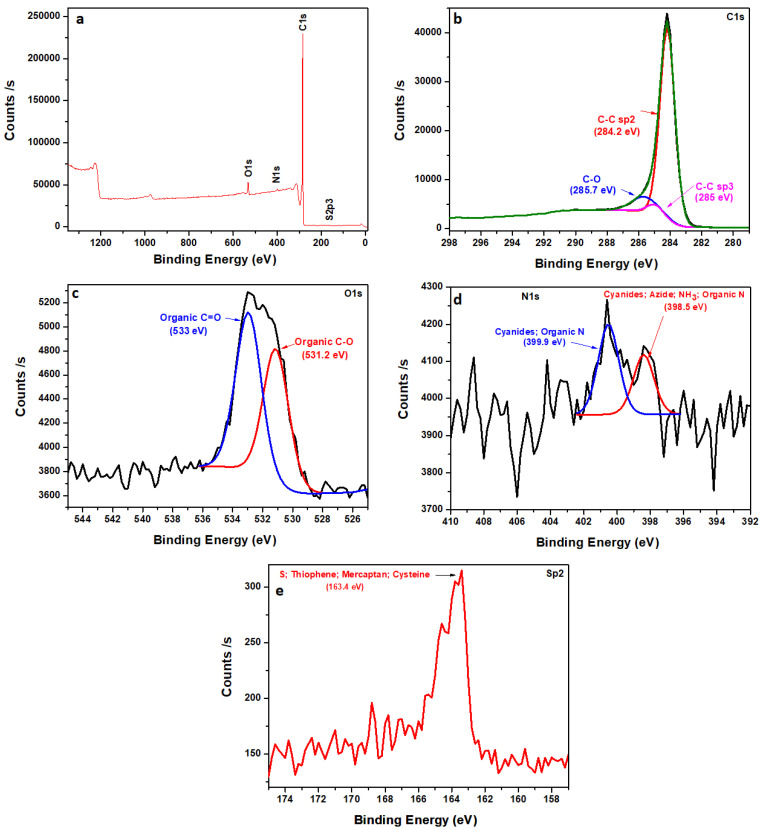
(**a**) XPS wide scan, (**b**) C 1s, (**c**) O 1s, (**d**) N 1s, and (**e**) Sp2 of NCF material.

Figure 5 shows an evaluation based on XPS analysis employed for understanding the NCF’s chemical composition. The wide scan XPS spectrum of the carbon is displayed in Figure 5a showing fractional concentrations of the atoms that make up the material’s major elements (C, N, and O) as indicated in the figure. Three significant peaks can be seen at distinct carbon atoms’ functional groups in Figure 5b, including the carbon bond; C-C (284.2 eV), epoxy group; C-O (285.7 eV), and another carbon bond; C-C (285 eV). The core level spectra of O1s are also shown in Figure 5c, with fitted peaks observable at approximately 533 (C=O), and 531.2 (C-O) that can be attributed to O 1s in C-O composition. In Figure 5d, two main peaks are observed at different functional groups for nitrogen atoms, which include 399.9, and 398.5 eV (N 1s). Figure 5e shows a major peak at around 163.4 eV (Sp2) for the sulphur functional group. This material’s described binding energies are consistent with those of other carbon-related materials described in the literature [12,25]. 

### 3.2. Electrochemical Characterisation

**Figure 6:**
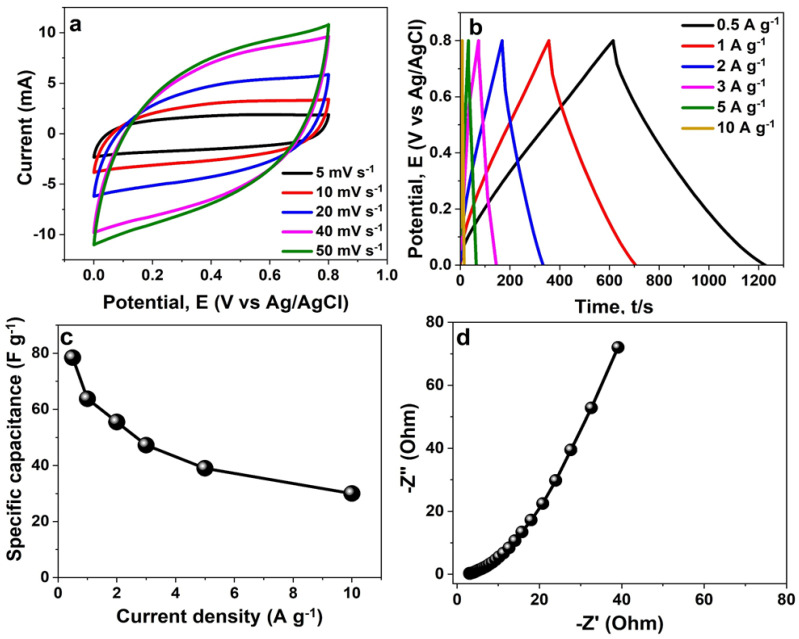
(**a**) CV, and (**b**) CD curves, (**c**) specific capacitance values for the NCF electrode at various scan rates, specific current, and current densities, respectively. (**d**) Plot of coulombic efficiency and capacitance retention against cycle numbers measured for the NCF material at 5 A g^−1^.

By using a three-electrode set-up and 2.5 M KNO_3_ as the study solution, the electrochemical characteristics of the as-synthesised NCF electrode were investigated. The related capacitive CV and GCD profiles for the electrode are depicted in Figure 6a,b for measurements between 0.0 V and 0.8 V potential window at various sweep rates and specific currents, accordingly. Figure 6c shows the electrode’s specific capacitance values recorded at various specific currents ranging from 0.5 to 10 A g^−1^. A maximum specific capacitance, *C_s_* calculated according to Equation (2) was recorded to be 78.4 F g^−1^ at 0.5 A g^−1^. The revealed specific capacitance values for the material as illustrated in Figure 6c could be attributed to the sample’s uniformly distributed interconnected nanofibre particles along with mesoporous structure as shown in Figure 1 and Figure 4b, respectively. It is evident that NCF material contains a high amount of S and O species (see Figure 3), which enhance the wettability of the electrode by the electrolyte. The EIS is a technique to further confirm the electrodes’ electrical properties. Figure 6d shows the Nyquist plots of the composites measured at an alternating potential of 10 mV in a frequency range of 100 kHz to 10 mHz. A short diffusion path length that is also near the ideal vertical line observed for the material suggests a fast ion diffusion rate and good electrochemical conductivity [26,31]. The resistance that results from the addition of the electrolyte’s ionic resistance, active materials’ intrinsic resistance, and resistance of contact at the interface of the active material and current collector [32,33,34,35], denoted as *R*_S_, is described by the intersection of the Nyquist plot and the horizontal axis. In Figure 6d, the *R*_S_ value of the electrode is estimated to be approximately 2.2 Ω. The observed low *R*_S_ value is accountable for the electrode’s good electrochemical performance, owing to its short diffusion path length ascribed to the fine size of the material’s porous structure.

**Figure 7:**
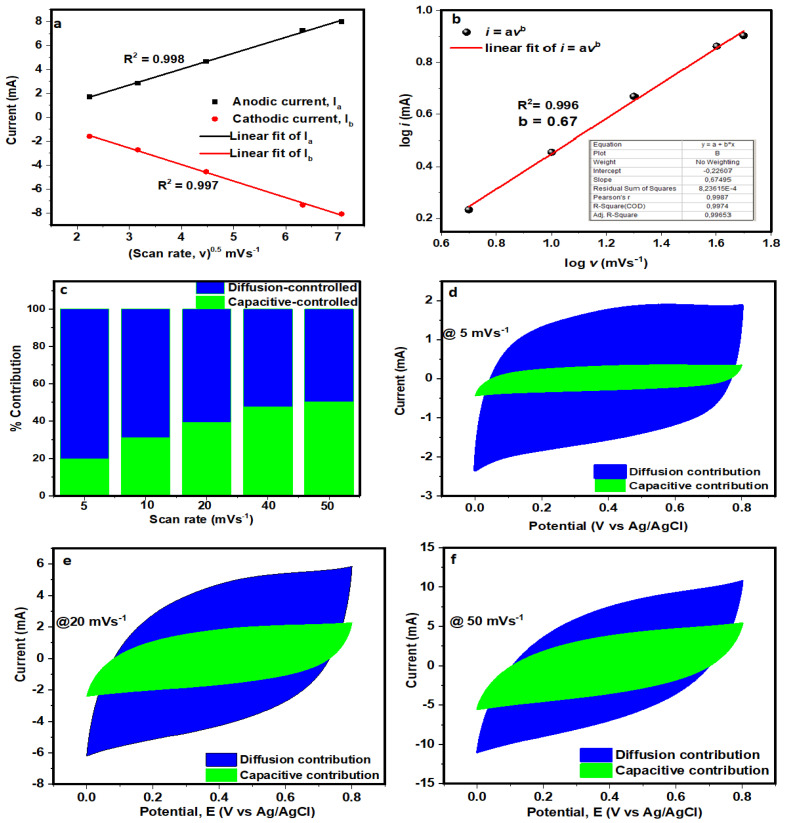
(**a**) *i*(*V*) vs. υ^1/2^ plots; (**b**) log (*i*) vs. log(υ) plot; (**c**) normalised contribution ratios of the capacitive and diffusion-controlled processes at different scan rates, and (**d**–**f**) CV curve at a scan rate of 5, 20, and 50 mV s^−1^, respectively.

By utilising the CV test at various scan rates within the range of 5–50 mV s^−1^, the electrochemical behavior and energy storage mechanism of the NCF electrode were thoroughly examined by the application of electrochemical kinetics studies. The relationship between the anodic and cathodic peak currents with scan rate in the electrode material establishes whether the specific capacitance originates from bulk diffusion or surface redox processes. Figure 7a depicts the plot of peak current (*i*) against the square root of the scan rate (υ^1/2^) for the electrode. The linear relationship suggests that the reaction is diffusion-controlled [36]. In addition, the R^2^ value of both the anodic (0.998) and cathodic (0.997) processes in the *i*-υ^1/2^ plot is almost 1, which suggests good capacitive properties and electrochemical reversibility of the electrode material. 

Moreover, the observed electrochemical reaction kinetics for the NCF electrode are further probed by the semiqualitative study of the correlation relationship between the *i* and *υ*, as shown in the following equations [37,38]:

(3) $$i=av^{b}$$

Equation (3) can be linearised to give

(4) log i=loga+b logv

where *a* and *b* are parameters that can be adjusted, and *i* and *υ* respectively represent peak current and scan rate. More specifically, the fitting line of *log i* vs. *log υ* in Figure 7b yielded the value of *b*. The optimal behavior of a surface capacitive-controlled redox process is established when the *b*-value approaches 1.0. In contrast, an ideal battery behavior is indicated when the *b*-value approaches 0.5 (diffusion-controlled redox reaction). In this study, the *b*-value for the electrode is 0.67, suggesting the existence of both surface- and diffusion-controlled charge storage mechanisms, with the latter being more prevalent. Furthermore, it is pertinent to calculate the contributions of the surface capacitive and diffusion-controlled procedures through Dunn’s method [39], respectively:

(5) $$i(V)={k_{1}v+k_{2}v}^{1/2}$$

The Equation (5) was rearranged and can be written as

(6) $$\frac{i\left(V\right)}{v^{1/2}}={k_{1}v}^{\frac{1}{2}}+k_{2}$$

where the current contributions from the surface capacitive and diffusion-controlled charges are denoted by *k*_1_*υ* and *k_2_υ*^1/2^, respectively. The fitting curve between i/υ^1/2^ and υ^1/2^ can be used to determine the values of *k*_1_ and *k*_2_. Figure 7c shows the capacitive contributions to the overall capacitance of the electrode at scan rates ranging from 5 to 50 mVs^−1^. At a scan rate of 5 mVs^−1^, as shown in Figure 7d, the diffusion effect contributes up to 80.3%, but the capacitive-controlled contribution is just 19.7%. Remarkably, given the finiteness of the ions’ intercalation into lattices at high scan rates, the capacitive-controlled process rapidly increases with increasing scan rates. Moreover, the capacitive-controlled contribution process was observed to have increased quickly for scan rates, indicating fast electrochemical kinetics at the electrode/electrolyte interface. A typical CV curve at 20 and 50 mV s^−1^ is shown in Figure 7e and Figure 7f, respectively, where the capacitive effect accounts for 39.1% and 50.3% of the total capacitance, while the diffusion effect accounts for 60.9% and 49.7%, respectively.

**Figure 8:**
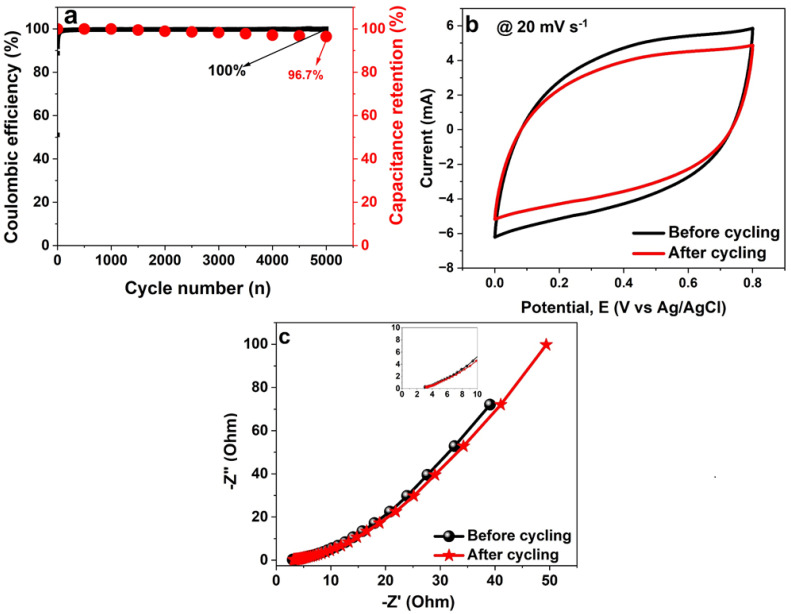
(**a**) cyclic performances, (**b**) CV curves before and after the cycling test, and (**c**) EIS Nyquist plot before and after 5000 cycles, respectively, for the NCF electrode at 5 A g^−1^.

The electrode as shown in Figure 8a has an exceptional coulombic efficiency of almost 100%, as estimated by Equation (2). It also preserves up to 96.7% of its original capacitance for more than 5000 cycles at 5 A g^−1^. A feature that is attributed to the networked and permeable structure is crucial in enhancing the effective electrode surface area and promoting electrolyte permeation. Consequently, decreasing the active materials’ electron pathways.

Figure 8b is a display of the nature of CV profiles for the NCF single electrode before and after being subjected to over 5000 GCD cycles at 5 A g^−1^. It can be noticed that after the long cycling test, the electrode’s current response marginally decreased, which is a correspondence of a rise in resistance [10]. In Figure 8c, the *R_s_* was observed to have increased from the original 2.2 Ω to 3.1 Ω after the 5000 GCD cycles.

The results obtained for the carbon nanofibres electrode in this work compare well with some similar materials already reported in the literature. Table 1 is a display of electrochemical properties of some similar carbon nanofibre electrodes tested in aqueous electrolytes compared with the NCF material in this work.

## 4. Conclusions

Using an easy, economical, and sustainable two-step synthesis approach, we have accomplished a successful synthesis and characterisation of PBI-based and electrospun carbon fibre (NCF) material in this work. The as-synthesised sample’s diverse material characterisation showed an interconnected nanofibre morphology with a material’s specific surface area (SSA) of about 49 m^2^ g^−1^. In addition, a promising electrochemical behaviour was shown by the three-electrode assembly, which produced a satisfactory electrochemical performance around a working potential of 0.8 V and a maximum specific capacitance value of 78.4 F g^−1^ at 0.5 A g^−1^ in 2.5 M KNO_3_ electrolyte. At a specified current of 5 A g^−1^, the electrode does not exhibit any significant capacitance loss (3.3%) of its initial value after a cycling test of more than 5000 cycles. Our findings point to the possibility of using this material to create high-performance energy storage devices with a room for improvement. More research is needed on materials based on carbon nanofibres in aqueous electrolytes for improved electrochemical performance in supercapacitor electrodes. The significant stability of the electrode in the selected electrolyte proved its potency as electrode material for applications in energy storage devices.

Future initiatives in this work will focus on creating novel approaches using surface functionalisation, hybridisation, and doping to improve the specific capacitance, rate capabilities, and cycling stability of the electrode. Additionally, the NCF’s electrochemical performance for use in supercapacitors may be further improved by combining it with other innovative materials, such as metal organic frameworks (MOFs) and conjugated microporous polymers (CMPs), and by investigating other electrolyte systems, like ionic liquids, organic, and solid electrolytes.

Electrospinning being a versatile and scalable technique used to fabricate nanofibres with controlled morphology and properties. The properties of the carbon nanofibres can be improved by adjusting the parameters of the electrospinning process. Several key parameters among others, can be optimised to enhance the properties of the carbon nanofibres. These include (i) higher polymer concentrations, (ii) proper balancing of viscosity and conductivity by adjusting solvent and additives, (iii) higher applied potential, (iv) higher flow rate of solution, (v) optimal collector distance, (vi) environmental conditions such as temperature and humidity that can affect solvent evaporation rates and fibre morphology, and (vii) introducing nanoparticles, carbon nanotubes, or graphene during electrospinning that can enhance electrical, thermal, and mechanical properties of the carbon nanofibres. 

By fine-tuning these parameters, researchers can tailor the diameter, surface morphology, porosity, and overall performance of carbon nanofibres to meet specific application requirements.

## Figures and Tables

**Table 1:** A comparative analysis of a few electrospun carbon nanofibre materials for use as supercapacitor electrodes.

Material	SSA (m^2^ g^−1^)	Specific Capacitance (F g^−1^)	Stability	Electrolyte	Reference
PBI/DMAc	1220	202 @ 1 mA cm^2^	N/A	1 M H_2_SO_4_	[18]
PAN-co-butadiene based CNF	N/A	172.0 @ 1 A g^−1^	100% after 2000 cycles	2 M KOH	[22]
PAN and MXene in DFM	N/A	205.0 mF cm^−2^ @ 50.0 mV/s	N/A	1 M H_2_SO_4_	[26]
PAN–DMSO/DMF	212	43 @ 2 mV s^−1^	N/A	1 M NaCl	[40]
PAN + biochar + DMF	30.12	37.60 @ 500.0 m (A g^−1^)	N/A	1 M NaOH	[41]
PAA–PDPP/DMF	130.6	182 @ 1 A g^−1^	88% after 10,000 cycles	6 M KOH	[42]
Porous CNFs	N/A	362.0 @ 0.2 A g^−1^	N/A	6 M KOH	[43]
hydroxyl-containing polyimide (HPI) nanofibres	1107.6	263.9 @ 0.5 A g^−1^	99.8% after 10,000 cycles	6 M KOH	[44]
PAN and 0.1 wt.% in N, N-dimethylformamide	684	10.0 @ 10.0 mV s^−1^	N/A	1 M KCl	[45]
CA–DMAc/acetone	1188	202 @ 0.1 A g^−1^,	92% after 5000 cycles	6 M KOH	[46]
PBI_NCFs	49	78 @ 0.5 A g^−1^	96.7% after 5000 cycles	2.5 M KNO_3_	This work

## Data Availability

The data adopted in support of the findings in this work are available upon request from the corresponding author.
